# High Expression of Cancer-Derived Glycosylated Immunoglobulin G Predicts Poor Prognosis in Pancreatic Ductal Adenocarcinoma

**DOI:** 10.7150/jca.39800

**Published:** 2020-02-03

**Authors:** Ming Cui, Lei You, Bang Zheng, Xinmei Huang, Qiaofei Liu, Jing Huang, Boju Pan, Xiaoyan Qiu, Quan Liao, Yupei Zhao

**Affiliations:** 1 Department of General Surgery, Peking Union Medical College Hospital, Chinese Academy of Medical Sciences & Peking Union Medical College, Beijing 100730, China; 2 School of Public Health, Faculty of Medicine, Imperial College London, London W6 8RP, UK; 3 Department of Immunology, School of Basic Medical Sciences, Peking University, Beijing 100191, China; 4 Peking University Center for Human Disease Genomics, Beijing 100191, China; 5 Department of Pathology, Peking Union Medical College Hospital, Chinese Academy of Medical Sciences & Peking Union Medical College, Beijing 100730, China

**Keywords:** pancreatic ductal adenocarcinoma, cancer-derived IgG, survival, prognosis

## Abstract

**Background**: Cancer-derived immunoglobulin G (CIgG) has been detected in various cancers and plays important roles in carcinogenesis. The present study aimed to investigate its clinical significance in pancreatic ductal adenocarcinoma (PDAC).

**Methods**: Using tissue microarrays (TMAs) and immunohistochemistry, we assessed CIgG expression in 326 patients who underwent surgical resection for PDAC. The associations between CIgG expression and clinicopathological features and clinical outcomes were analyzed. Functional experiments were also performed to investigate the effect of CIgG on PDAC cells.

**Results**: High CIgG expression was related to poor tumor differentiation and metastasis during follow-up and was associated with poor disease-free survival (DFS) and overall survival (OS). A multivariate Cox regression analysis identified high CIgG expression as an independent prognostic factor for DFS and OS. The incorporation of CIgG expression improved the accuracy of an established prognosis prediction model for 1-year OS and 2-year OS. *In vitro* studies showed that knocking down CIgG profoundly suppressed the proliferation, migration, and invasion capacity of PDAC cells.

**Conclusions**: CIgG contributes to the malignant behaviors of PDAC and offers a powerful prognostic predictor for these patients.

## Introduction

Pancreatic ductal adenocarcinoma (PDAC), the most common histological type that accounts for more than 90% of pancreatic cancer cases, remains one of the most lethal malignancies 1. The incidence of pancreatic cancer has increased yearly; however, the five-year survival rate remains less than 10% 2. Pancreatic cancer has been predicted to become the second leading cause of cancer-associated mortality within the next 10 years 3. PDAC is a highly complex and heterogeneous malignancy with distinct prognoses and responses to treatment 4. In recent years, the prognostic values of an increasing number of molecular markers have been assessed in PDAC, but the number of powerful prognostic markers remains limited 5.

Immunoglobulins (Igs) are a well-known family of classic immune molecules that play important roles in humoral immunity responses. In recent years, Ig expression has been widely found in a wide range of tumor cells 6-12. Furthermore, increasing evidence has revealed that cancer-derived immunoglobulin G (CIgG) displays growth factor-like activity, promotes tumor growth and metastasis, and indicates poor prognosis in patients with cancer 10,13,14. Importantly, IgG has been detected in human PDAC tissue and cell lines and was indicated to play a role in promoting tumor growth 15,16. However, the clinical significance of CIgG in PDAC diagnosis, prognosis prediction, and therapy remains unclear.

In previous studies, commercial antibodies against human IgG have been used to detect IgG expression in PDAC 15,16; however, these antibodies cannot distinguish between CIgG and B cell-derived IgG (B-IgG). RP215 is a monoclonal antibody originally raised to specifically recognize cancer-associated antigens 17. The antigen recognized by RP215 was later validated to be CIgG expressed in various epithelial human cancers 18,19. Further studies showed that RP215 recognizes a glycosylated epitope involving a noncanonical N-glycosylation modification of CIgG that distinguishes CIgG from B-IgG 12. This glycosylated IgG is highly expressed in cancer stem cells and promotes cancer initiation and metastasis in epithelial cancer 20. Importantly, RP215 shows potential therapeutic effects in epithelial cancer by directly recognizing and blocking the glycosylation modification of CIgG *in vitro* and *in vivo* 12,21.

In this study, we investigated the correlation between CIgG and clinicopathological factors and examined the prognostic impact of CIgG in more than 300 PDAC cases for the first time. Tumor-infiltrating B cells (B-TILs) were also detected in the study. Moreover, the molecular function of CIgG in PDAC cells was evaluated by *in vitro* experiments.

## Materials and Methods

### Patients and study design

A cohort of 381 patients with a diagnosis of PDAC who underwent curative surgery (tumor margin >1 mm) at Peking Union Medical College Hospital between 2004 and 2014 were assessed for eligibility. Patients were excluded according to the following criteria: preoperative chemotherapy and/or radiotherapy, pathological diagnosis other than PDAC, and perioperative death. After exclusion, 326 archived formalin-fixed, paraffin-embedded (FFPE) PDAC tumor and adjacent nontumor pancreatic tissue samples were examined. The staging was based on the 7^th^ edition Staging Manual of the American Joint Committee on Cancer (AJCC). The median follow-up time was 18 (range 1-129) months. This study was approved by the medical ethics committee at Peking Union Medical College Hospital (S-K 623). All the patients enrolled in this study provided written informed consent.

### Tissue microarray (TMA) construction and immunohistochemical staining

TMAs were constructed by a manual tissue arrayer (Beecher Instruments, Sun Prairie, WI, USA) using FFPE blocks. Tumor and adjacent nontumor tissue cores from each patient were harvested from representative areas using a 1.5-mm tissue punch. The monoclonal antibody RP215 was used to specifically recognize glycosylated CIgG. A commercial rabbit anti-human IgG polyclonal antibody (269A-16, Cell Marque, CA, USA) was also used to detect IgG in the PDAC samples; however, this antibody extensively stained IgG in lymphocytes, normal pancreatic cells, and cancer cells (Fig. S1). Due to the much lower specificity of the commercial antibody in recognizing CIgG, the monoclonal antibody RP215 (5 μg/ml) was ultimately used. A mouse anti-human CD20 monoclonal antibody (0.16 μg/m; NCL-L-CD20-L26, Leica) was used to recognize B cells. Immunohistochemistry was performed as described previously 10.

### Evaluation of the immunohistochemical results

After staining, the TMA slides were digitalized using Panoramic MIDI (3D HISTECH, Hungary). The staining evaluation was independently performed by two independent investigators (M.C. and B.P.) who were blinded to the patient clinical outcomes. An H-score was applied for evaluation of CIgG expression 22. The absolute number of intratumoral CD20-positive B cells was determined to reveal the number of B-TILs. Each TMA slide core was divided into 6 equal parts. CIgG expression and the B-TIL count were evaluated in a high-powered field (400× magnification). The average value of the count in all 6 parts was considered the representative value of the patients. Optimal cutoff values of 148 for CIgG expression and 2 cells/high-powered field for the B-TIL count were determined to predict prognosis using X-tile 3.6.1 software (Yale University, New Haven, CT, USA) 23.

### Cell culture

BxPC-3, T3M4, AsPC-1, CFPAC-1, PANC-1 and HPAF PDAC cell lines were purchased from the American Type Culture Collection (ATCC, Manassas, USA) and cultured in RPMI-1640/DMEM supplemented with 10% FBS and 1% antibiotics at 37 ℃ under 5% CO2. Fibroblast cell lines (CAF19 and SC2) were generously provided by Dr. Jun Yu (Department of Surgery, The Johns Hopkins University School of Medicine, Baltimore, MD, USA) and cultured in DMEM supplemented with 10% FBS and 1% antibiotics at 37 ℃ under 5% CO2.

### Western blot

Western blot assays were performed as described previously 10. The primary antibodies used for Western blotting were as follows: RP215 (0.5 μg/ml) and GAPDH (1:1,000; H-12, Santa Cruz, CA, USA).

### Immunofluorescence

Immunofluorescence was performed as reported previously 11. RP215 was used as the primary antibody (5 μg/ml) and the goat anti-mouse IgG (H+L) secondary antibody Alexa Fluor 488 (Invitrogen, A-11001) was used as the secondary antibody. Nuclei was stained by DAPI. Images were captured via a confocal laser scanning microscope (Nikon A1R).

### RT-PCR and analysis of variable region sequence in rearranged IgHγ in PDAC cells

Nested PCR for variable region sequencing (V_Hγ_D_γ_J_Hγ_ sequence) of rearranged IgHγ in BxPC-3 cells was performed as reported previously 8. The PCR products were then cloned into the pGEM-T Easy Vector (Promega) and sequenced with an ABI 3100 DNA sequencer (Thermo Fished Scientific). The V_Hγ_D_γ_J_Hγ_ sequence was then analyzed with the Basic Local Alignment Search Toll (BLAST).

### Small interfering (si) RNA transfection

siRNAs targeting the constant region of the IgHγ-chain (siRNA1: 5'-GGUGGACAAGACAGUUGAG-3', siRNA2:5'-AGUGCAAGGUCUCCAACAA-3', and nonsilencing control RNA (NC): 5'-UUCUCCGAACGUGUCACGU-3') were transfected into BxPC-3 and T3M4 cells with Lipofectamine^TM^ 3000 (Invitrogen) according to the manufacturer's protocol.

### Cell proliferation assay

Forty-eight hours after siRNA transfection, cells were collected and plated in 96-well plates at a density of 2,000 cells per well. A CCK8 (Cell Counting Kit-8, CK04, Dojindo) assay was used to analyze cell proliferation on days 0, 1, 2, 3, and 4. After 2 hours incubation with CCK8 reagent, the number of viable cells was recorded by measuring the absorbance at 450 nm.

### Colony formation assay

Forty-eight hours after siRNA transfection, cells were collected and seeded into 12-well plates at a density of 200 cells per well. After culturing for ten days, cells were stained with 1% crystal violet, and colonies were counted.

### Cell migration and invasion assay

Migration and invasion assays were performed in 24-well transwell plates (Corning) containing a polycarbonate membrane (8.0 μm pore size). The chambers were precoated with Matrigel (Corning) for the invasion assays. Forty-eight hours after siRNA transfection, cells were collected and seeded onto the upper surface of the transwell membrane. After 24 hours, the nonmigrating cells from the upper surface were removed, and the cells that had migrated to the bottom of the chambers were stained with 1% crystal violet and counted in 6 randomly selected fields under a microscope.

### Statistical analysis

A χ^2^ test was used for categorical variables, and a t test was used for continuous variables. The levels of CIgG in the tumor and peritumoral tissue samples were compared using a Wilcoxon signed-rank test. Spearman's rank correlation coefficients between CIgG expression and the B-TIL count were tested. Kaplan-Meier survival curves were generated after stratification of the data and compared with a log-rank test. The Cox proportional hazards model was used to examine associations between clinicopathological features and disease-free survival (DFS) or overall survival (OS). The discrimination and calibration properties of the prediction model derived from the cohort were assessed with the C-index and a calibration plot. Moderating effects of other clinicopathological features on the prognostic value of CIgG were tested in Cox regression models by adding interaction terms, with adjustments for sex and age group. The statistical analyses were performed using IBM SPSS 24.0, GraphPad Prism 6, and STATA 14 software. All statistical tests were two-sided, and the significance level was defined as *P*<0.05.

## Results

### CIgG expression, the B-TIL count, and associations with clinicopathological features

**Figure 1:**
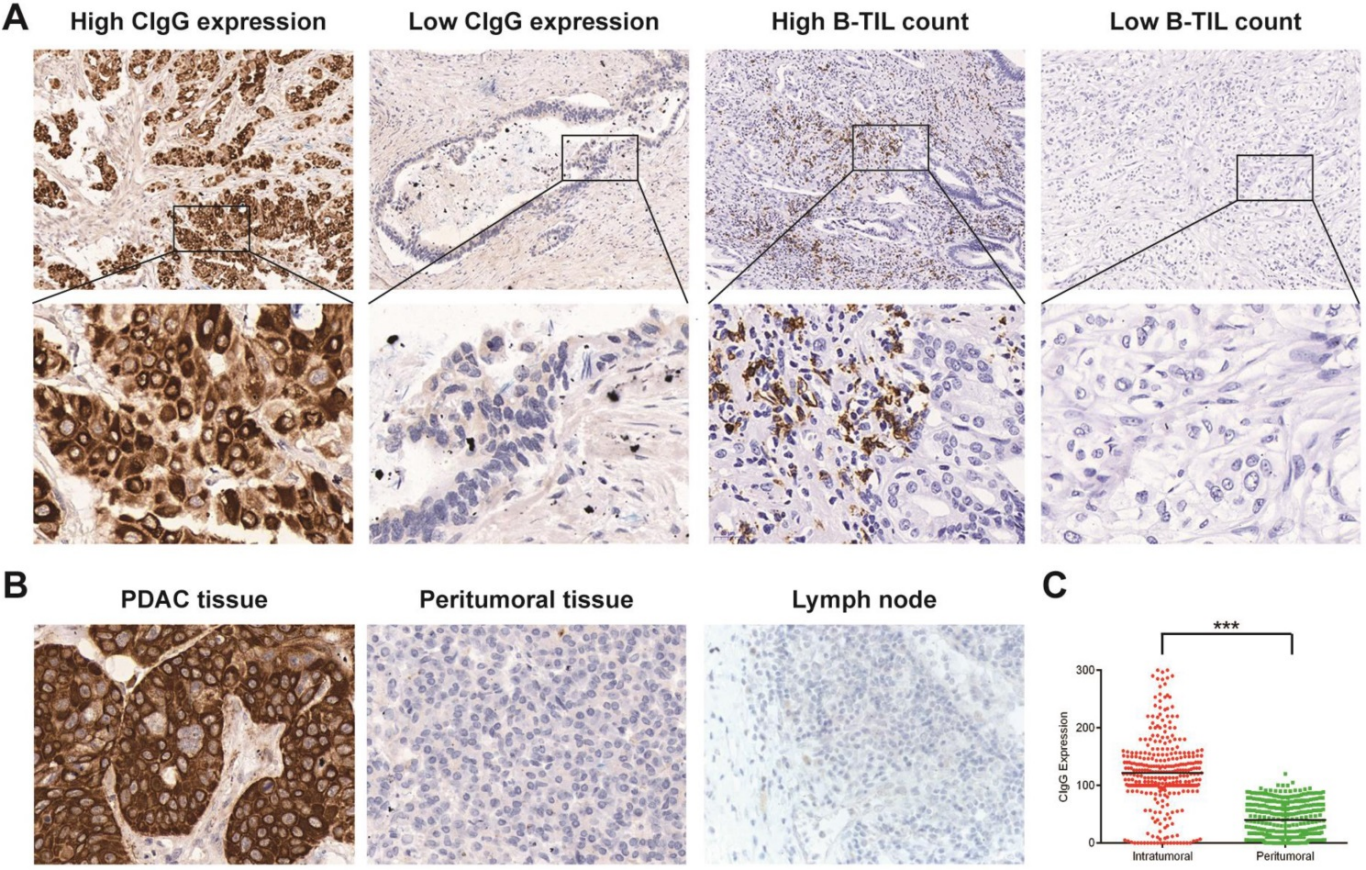
** Staining of CIgG and B-TILs in PDAC tissues.** (A) Representative microphotographs of CIgG and B-TIL staining in PDAC tissue; Original magnification, 100× (upper panels) or 400× (lower panels). (B) Representative microphotographs showing specific staining of CIgG in the tumor cells of PDAC tissue compared to peritumoral tissue as well as lymph nodes; Original magnification, 400×. (C) Intratumoral and peritumoral CIgG expression was compared in each patient. ***P<0.001.

CIgG staining was positive in 283 patients (86.8%). According to the optimal cutoff value of CIgG expression, CIgG was highly expressed in 91 patients (27.9%) (Fig. 1A). Notably, CIgG was highly expressed specifically in the tumor cells (Fig. 1B). The expression of CIgG was significantly higher in the PDAC tumor tissue than in the peritumoral tissue (*P*<0.001) (Fig. 1C). Staining for B-TILs was positive in 158 patients (48.5%), and high B-TIL counts were detected in 80 patients (24.5%) based on the optimal cutoff value (Fig. 1A). CIgG expression was not correlated with the B-TIL count (*r_s_*=0.008, *P*=0.885, Fig. S2). High CIgG expression was significantly associated with poor tumor differentiation (*P*<0.001) and metastasis during follow-up (*P*=0.006). There were no significant differences in other variables between the high CIgG expression group and the low CIgG expression group (Table 1). There were no significant differences in the studied variables between the high B-TIL count group and the low B-TIL count group (Table 1).

### CIgG expression, the B-TIL count, and prognosis in patients with PDAC

**Figure 2:**
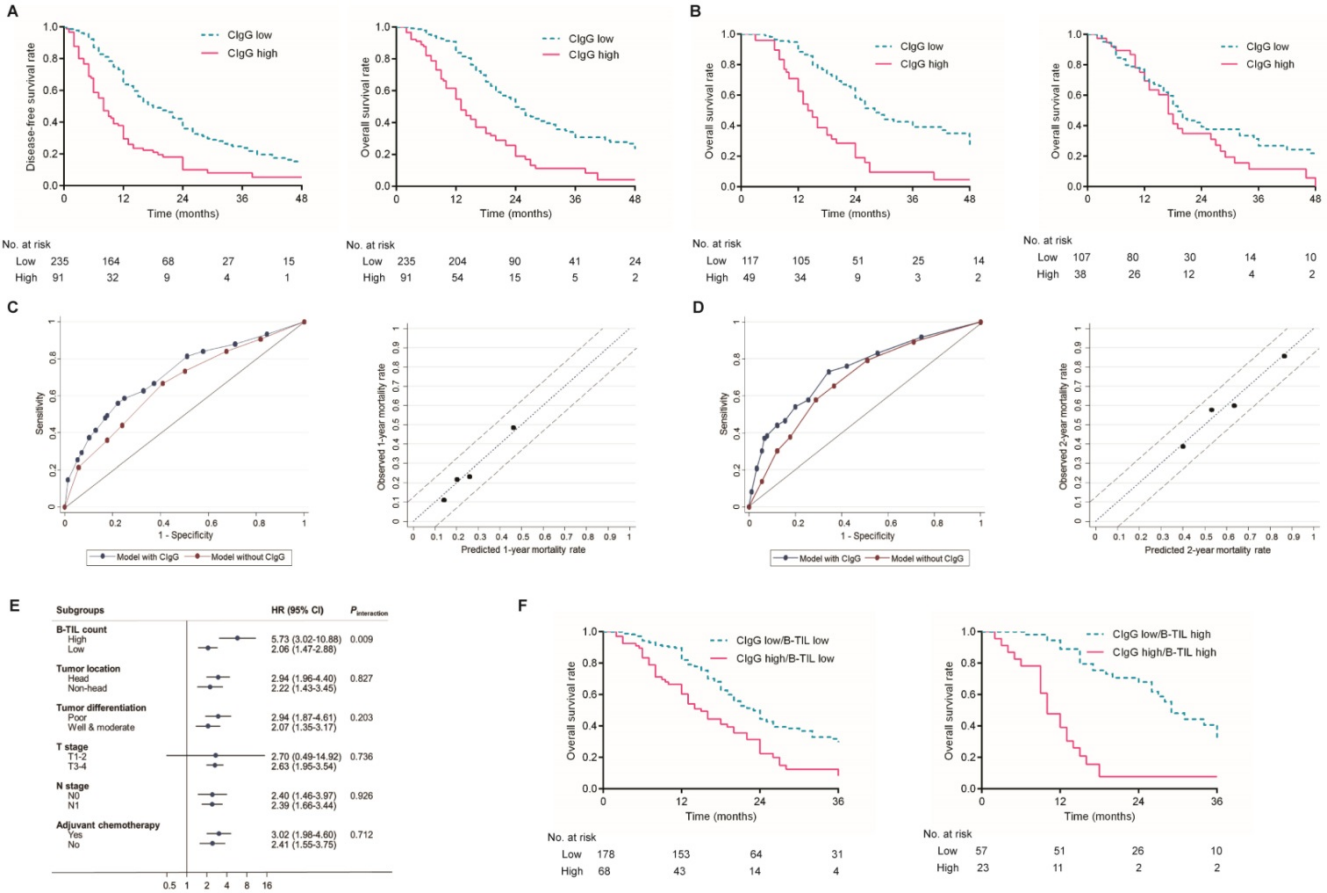
** Prognostic value of CIgG expression in PDAC.** (A) Kaplan-Meier curves for DFS and OS based on the expression of CIgG. (B) Kaplan-Meier curves for OS based on the expression of CIgG in PDAC patients receiving adjuvant chemotherapy (left) or not (right). (C, D) ROC curve and calibration plot of the prediction model for 1-year/2-year prognosis and mortality rate. (E) Subgroup analysis of associations between CIgG expression and OS risk according to clinicopathologic factors. (F) Kaplan-Meier curves for OS according to CIgG expression and the B-TIL count.

Kaplan-Meier analysis showed that patients with high CIgG expression experienced significantly shorter DFS and OS than those with low CIgG expression (median DFS: 8 months vs. 18 months, respectively, *P*<0.001; median OS: 13 months vs. 24 months, respectively, *P*<0.001, Fig. 2A). A high B-TIL count was not shown to be prognostic for DFS or OS (Fig. S3). Notably, high CIgG expression was significantly associated with worse survival among PDAC patients who received adjuvant chemotherapy (*P*<0.001) but not associated with worse survival among PDAC patients who did not receive adjuvant chemotherapy (*P*=0.100) (Fig. 2B). Univariate and multivariate analyses for DFS and OS are shown in Table 2. CIgG expression was identified as an independent prognostic factor associated with both DFS and OS by the multivariate Cox regression analysis.

### Evaluation of the discrimination and calibration of a prediction model for OS

Prediction models for OS risk were derived from the multivariate Cox model (Table 2). The discriminative accuracy of a model utilizing CIgG expression, N stage, tumor differentiation, and adjuvant chemotherapy as predictors was compared with that of a model without CIgG expression. The C-index for the 1-year OS outcomes was significantly improved after inclusion of CIgG expression in the prediction model (0.716 with CIgG expression vs. 0.658 without CIgG expression, *P*=0.036). A similar significant improvement in the C-index was observed for the prediction of 2-year OS outcomes (0.736 with CIgG expression vs. 0.685 without CIgG expression, *P*=0.025). The calibration plot was generated for the full prediction model in terms of the 1-year and 2-year mortality risks. The predicted 1-year and 2-year mortality rates agreed perfectly with the observed mortality rates (Fig. 2C and D).

### B-TILs potentially contributed to the unfavorable prognostic value of CIgG expression in PDAC

The interactions between CIgG expression and other clinicopathological variables in regard to the OS risk were further tested using Cox regression models. Interestingly, a high B-TIL count showed a significant synergistic effect with CIgG expression (*P_interaction_*= 0.009, Fig. 2E), while no other variables exhibited a moderating effect. Kaplan-Meier curves showed that the effect of CIgG expression on the OS risk was more evident in patients with high B-TIL counts than in those with low B-TIL counts (Fig. 2F). The results of the Cox regression analysis demonstrated that compared with patients with low B-TIL counts, who had CIgG HRs of 2.001, the patients with high B-TIL counts showed a stronger unfavorable prognostic value for high CIgG expression (HR=4.495).

### CIgG promoted the proliferation, migration, and invasion capacity of PDAC cells

**Figure 3:**
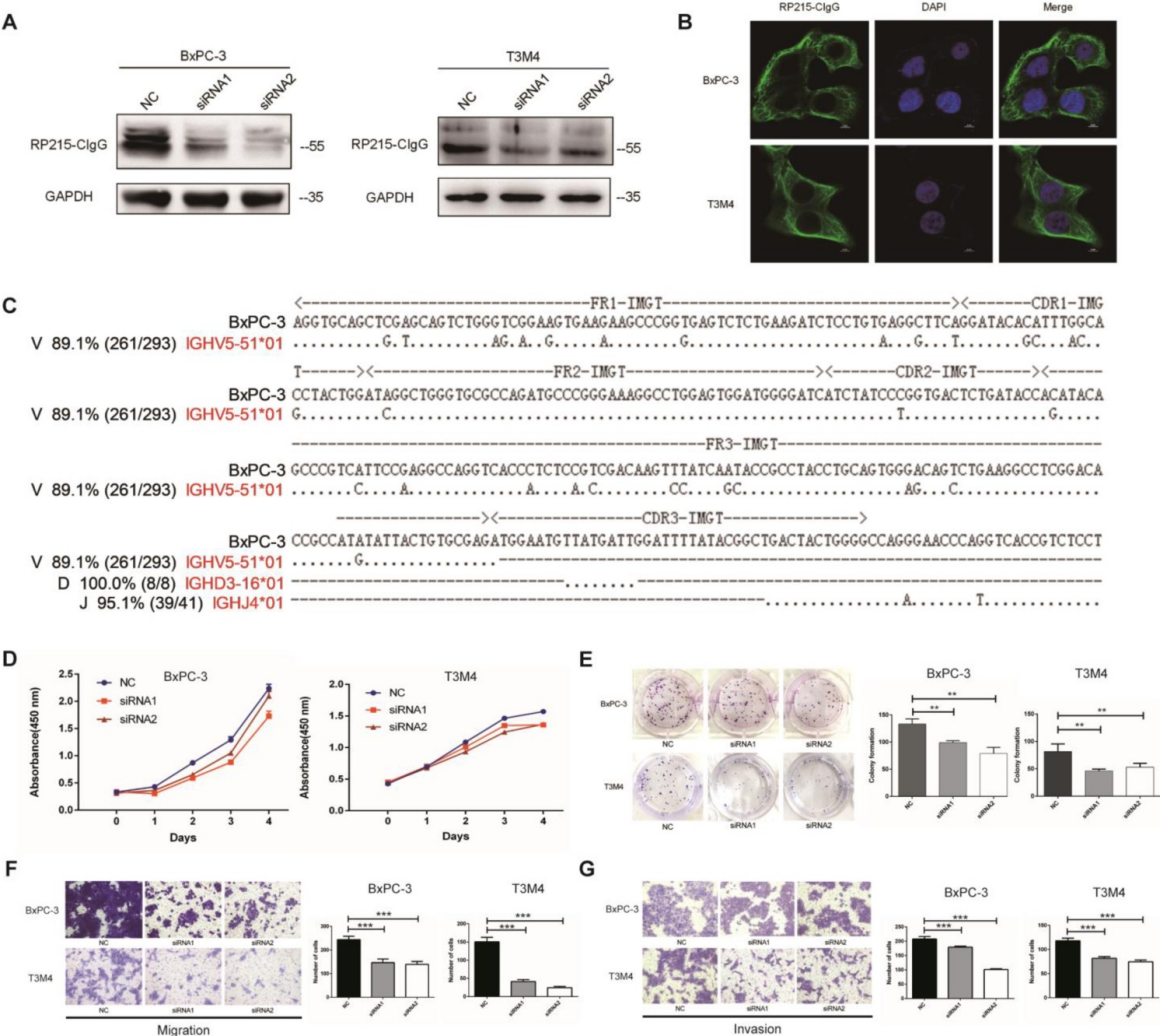
** CIgG promoted the proliferation, migration, and invasion capacity of PDAC cells.** (A) CIgG expression and CIgG knockdown by siRNAs in BxPC-3 and T3M4 cells. (B) Localization of CIgG in BxPC-3 and T3M4 cells. (C) Recombinant V_Hγ_D_γ_J_Hγ_ sequence amplified from BxPC cells. Dots, identical sequences; capital letters, mutations. (D) Cell viability of BxPC-3 and T3M4 cells treated with siRNA was measured by CCK8 assays. (E) BxPC-3 and T3M4 cells treated with siRNA were subjected to colony formation assay. Representative images are shown for each group. (F) BxPC-3 and T3M4 cells treated with siRNA were subjected to migration assay. Representative images are shown for each group. Original magnification, 200×. (G) BxPC-3 and T3M4 cells treated with siRNA were subjected to invasion assay. Representative images are shown for each group. Original magnification, 200×. **P<0.01, ***P<0.001.

CIgG expression was detected in BxPC-3, T3M4, AsPC-1, CFPAC-1, PANC-1 and HPAF PDAC cell lines as well as fibroblasts by Western blotting (Fig. 3A and Fig. S4). CIgG was localized on the cell membrane and in the cytoplasm of PDAC cells (Fig. 3B). Rearranged transcripts of the variable region sequence of IgHγ in BxPC-3 cells were further amplified and analyzed. Recombination of the V_Hγ_D_γ_J_Hγ_ variable region in BxPC-3 cells showed a restrictive pattern of IGHV5-51/IGHD3-16/IGHJ4 (Fig. 3C). Furthermore, we investigated the function of CIgG in PDAC cells using siRNA to knock down CIgG expression (Fig. 3A). The CCK8 assay showed that knockdown of CIgG decreased the viability of BxPC-3 and T3M4 cells (Fig. 3D). The colony formation assay showed that BxPC-3 and T3M4 cells formed fewer clones after CIgG knockdown (Fig. 3E). Finally, the migration and invasion assay showed that CIgG knockdown significantly suppressed the migration and invasion capability of BxPC-3 and T3M4 cells (Fig. 3F and G).

## Discussion

In this study, we investigated the prognostic value of CIgG expression in 326 patients with PDAC. Our results showed that CIgG was specifically highly expressed in PDAC. High expression of CIgG was correlated with poor differentiation and metastasis during follow-up in PDAC patients. More importantly, CIgG expression was shown to be a powerful prognostic marker for survival.

IgG expression has been detected in various tumor cells, known as CIgG, and has been shown to be correlated with worse prognosis in patients with cancer 6,10,11. Many studies have focused on the underlying mechanisms involving CIgG in carcinogenesis. One possible mechanism is that CIgG exhibits growth-factor-like activity, promoting proliferation, invasion, and metastasis in cancer cells 6, 10. A recent study further showed that CIgG can execute its oncogenic function by interacting with the integrin α6β4 complex and activating the FAK and Src pathways 12. Another possible mechanism is that CIgG could regulate the functions of immune cells and mediate immune escape 15,24. In the present study, the expression of CIgG was validated specifically in PDAC cell lines. *In vitro* functional experiments showed that CIgG promoted the proliferation, invasion, and migration capacity of PDAC cells.

The significance of B-TILs in PDAC was also investigated in our study. B-TILs have been found to have dual effects of tumor promotion and suppression 25-26. In our study, the B-TIL count was not correlated with CIgG expression, which is understandable since it has been validated that CIgG is derived from tumor cells. In addition, the B-TIL count was not associated with the prognosis of patients with PDAC. Interestingly, compared with patients with low B-TIL counts, a relatively stronger unfavorable prognostic value for the high expression of CIgG in patients with high B-TIL counts was observed, indicating a synergistic effect between B-TILs and CIgG in PDAC carcinogenesis. The mechanisms underlying this phenomenon remain unclear. One possible explanation might be that the cytokines released by B-TIL facilitate the production of CIgG in tumor cells.

Emerging evidence has shown that combination treatments with standard chemotherapy plus novel drugs targeting tumor cell-autonomous signaling pathways or tumor microenvironment might be promising therapies for PDAC 27. RP215 is a monoclonal antibody that has been validated to specifically recognize glycosylated CIgG 12,17,18. RP215 treatment can induce apoptosis in cancer cells *in vitro* and control the growth of tumors *in vivo* 12,21. In our study, we found that nearly 30% of the PDAC patients presented high CIgG expression, which indicates that targeting CIgG with the RP215 antibody might have a therapeutic effect. Furthermore, our results showed that patients with high CIgG expression who received postoperative adjuvant chemotherapy exhibited worse survival, and thus, therapies targeting CIgG might hold promise for this subgroup of patients who may not obtain benefits from classical chemotherapy.

One limitation of the present study is its retrospective design. Prospective studies with the enrollment of more centers may validate CIgG's prognostic value in PDAC. In addition, the molecular mechanism for CIgG in the carcinogenesis of PDAC is still unclear. Furthermore, preclinical research is needed to explore the therapeutic effect and possible underlying mechanisms of the RP215 antibody against PDAC.

## Conclusion

In summary, our study revealed that CIgG expression was associated with clinical outcomes and could serve as a powerful prognostic factor in PDAC. Considering the dilemma of treatment for PDAC, these findings might pave the way for the application of CIgG-targeting therapies to improve the prognosis of this stubborn malignancy.

## Supplementary Material

Supplementary figures.

## Figures and Tables

**Table 1:** CIgG expression, B-TIL count and clinicopathological features

Clinicopathological Features	Total (n)	CIgG Expression		*P*	B-TIL Count		*P*
		Low	High		Low	High	
**All cases**	326	235 (72.1%)	91 (27.9%)		246 (75.5%)	80 (24.5%)	
**Age (y), mean±SD**	60.1±9.6	59.6±9.6	61.2±9.5	0.202	59.9±10.0	60.6±8.3	0.603
**Sex**				0.604			0.142
Male	186	132 (71.0%)	54 (29.0%)		146 (78.5%)	40 (21.5%)	
Female	140	103 (73.6%)	37 (26.4%)		100 (71.4%)	40 (28.6%)	
**Tobacco use**				0.234			0.507
Yes	116	79 (68.1%)	37 (31.9%)		90 (77.6%)	26 (22.4%)	
No	210	156 (74.3%)	54 (25.7%)		156 (74.3%)	54 (25.7%)	
**Alcohol use**				0.059			0.327
Yes	61	38 (62.3%)	23 (37.3%)		49 (80.3%)	12 (19.7%)	
No	265	197 (74.3%)	68 (25.7%)		197 (74.3%)	68 (25.7%)	
**Tumor location**				0.098			0.282
Head	192	145 (75.5%)	47 (24.5%)		149 (77.6%)	43 (22.4%)	
Non-head	134	90 (67.2%)	44 (32.8%)		97 (72.4%)	37 (27.6%)	
**Tumor differentiation**				**<0.001**			0.146
Well & moderate	206	165 (80.1%)	41 (19.9%)		150 (72.8%)	56 (27.2%)	
Poor	120	70 (58.3%)	50 (41.7%)		96 (80.0%)	24 (20.0%)	
**T stage**				0.081			0.854
T1-2	79	63 (79.7%)	16 (20.3%)		59 (74.7%)	20 (25.3%)	
T3-4	247	172 (69.6%)	75 (30.4%)		187 (75.7%)	60 (24.3%)	
**N stage**				0.060			0.767
N0	138	107 (77.5%)	31 (22.5%)		103 (74.6%)	35 (25.4%)	
N1	188	128 (68.1%)	60 (31.9%)		143 (76.1%)	45 (23.9%)	
**Metastasis during follow-up**				**0.006**			0.640
Yes	150	97 (64.7%)	53 (35.3%)		115 (76.7%)	35 (23.3%)	
No	176	138 (78.4%)	38 (21.6%)		131 (74.4%)	45 (25.6%)	
**Adjuvant chemotherapy**				0.516			0.535
Yes	166	117 (70.5%)	49 (29.5%)		122 (73.5%)	44 (26.5%)	
No	145	107 (73.8%)	38 (26.2%)		111 (76.6%)	34 (23.4%)	
Unknown	15						

**Table 2:** Cox regression analysis for disease-free survival and overall survival

**Variables for Disease-free Survival**	**Univariate Analysis**			**Multivariate Analysis**	
	**HR (95% CI)**	***P* value**		**HR (95% CI)**	***P* value**
Age≥65 years (vs <65 years)	0.857 (0.654-1.123)	0.262		-	-
Female (vs male)	0.904 (0.698-1.170)	0.443		-	-
Tumor location (non-head vs head)	0.924 (0.713-1.199)	0.553		-	-
Tumor differentiation (poor vs well & moderate)	1.477 (1.138-1.915)	**0.003**		1.381 (1.054-1.809)	**0.019**
T stage (T3-4 vs T1-2)	1.335 (0.987-1.807)	0.061		-	-
N stage (N1 vs N0)	1.446 (1.112-1.880)	**0.006**		1.406 (1.071-1.846)	**0.014**
Adjuvant chemotherapy (yes vs no)	0.917 (0.705-1.193)	0.521		-	-
CIgG expression (high vs low)	2.355 (1.786-3.106)	**<0.001**		2.359 (1.767-3.151)	**<0.001**
B-TIL count (high vs low)	1.094 (0.813-1.472)	0.554		-	-
**Variables for Overall Survival**	**Univariate Analysis**			**Multivariate Analysis**	
	**HR (95% CI)**	***P* value**		**HR (95% CI)**	***P* value**
Age ≥65 years (vs <65 years)	0.855 (0.642-1.140)	0.286		-	-
Female (vs male)	0.906 (0.689-1.190)	0.478		-	-
Tumor location (non-head vs head)	1.004 (0.763-1.322)	0.975		-	-
Tumor differentiation (poor vs well & moderate)	1.672 (1.271-2.200)	**<0.001**		1.522 (1.143-2.026)	**0.004**
T stage (T3-4 vs T1-2)	1.447 (1.040-2.012)	**0.028**		-	-
N stage (N1 vs N0)	1.684 (1.273-2.228)	**<0.001**		1.503 (1.124-2.011)	**0.006**
Adjuvant chemotherapy (yes vs no)	0.782 (0.591-1.033)	0.083		0.745 (0.563-0.986)	**0.040**
CIgG expression (high vs low)	2.550 (1.907-3.411)	**<0.001**		2.490 (1.830-3.387)	**<0.001**
B-TIL count (high vs low)	0.927 (0.673-1.276)	0.640		-	-
