# High Expression of Cancer-Derived Glycosylated Immunoglobulin G Predicts Poor Prognosis in Pancreatic Ductal Adenocarcinoma: Erratum

**DOI:** 10.7150/jca.32930

**Published:** 2021-09-05

**Authors:** Ming Cui, Lei You, Bang Zheng, Xinmei Huang, Qiaofei Liu, Jing Huang, Boju Pan, Xiaoyan Qiu, Quan Liao, Yupei Zhao

**Affiliations:** 1 Department of General Surgery, Peking Union Medical College Hospital, Chinese Academy of Medical Sciences & Peking Union Medical College, Beijing 100730, China; 2 School of Public Health, Faculty of Medicine, Imperial College London, London W6 8RP, UK; 3 Department of Immunology, School of Basic Medical Sciences, Peking University, Beijing 100191, China; 4 Peking University Center for Human Disease Genomics, Beijing 100191, China; 5 Department of Pathology, Peking Union Medical College Hospital, Chinese Academy of Medical Sciences & Peking Union Medical College, Beijing 100730, China

In the original version of the article, the representative image of siRNA2-silenced T3M4 cells in Figure 3G is incorrect. The correct Figure 3G is as follows. This correction will not affect the results and conclusions. The authors would like to apologize for any inconvenience this may have caused.

## Figures and Tables

**Figure 3:**
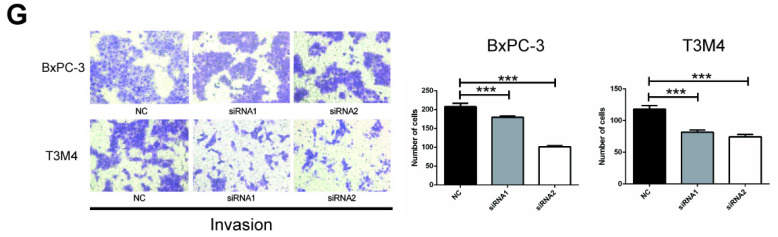
(G) BxPC-3 and T3M4 cells treated with siRNA were subjected to invasion assay. Representative images are shown for each group. Original magnification, 200×. **P<0.01, ***P<0.001.
