# Supplementary material for: High Expression of Cancer-Derived Glycosylated Immunoglobulin G Predicts Poor Prognosis in Pancreatic Ductal Adenocarcinoma
Source: J Cancer. 2020 Feb 3;11(8):2213–21. doi: 10.7150/jca.39800 (PMC7052941; doi:10.7150/jca.39800)
Supplement: Supplementary file 1 — Supplementary figures. [file jcav11p2213s1.pdf]

Supplemental data

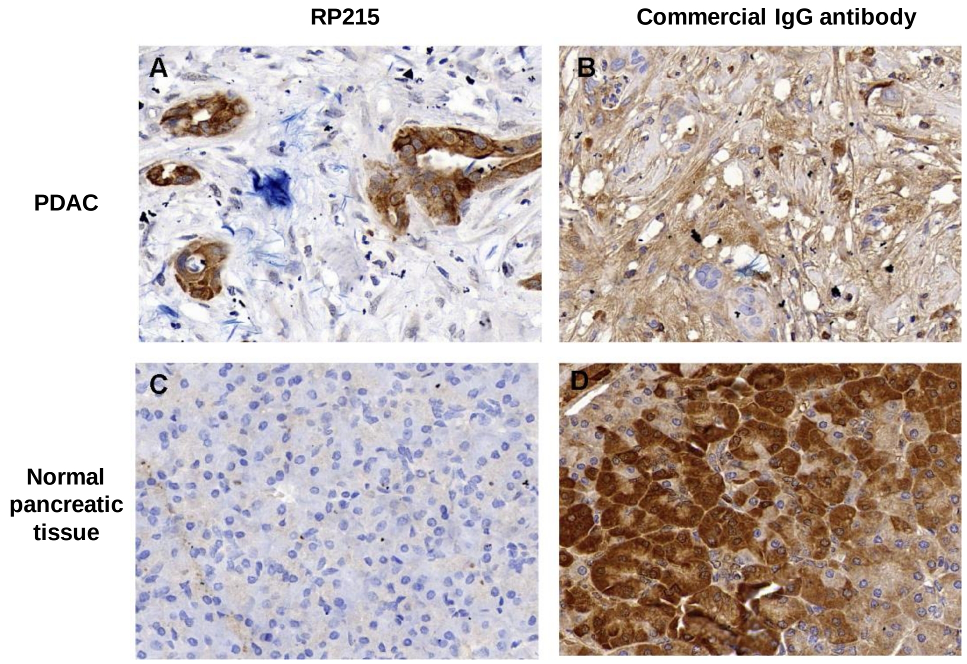

Fig. S1. Compared with RP215, which specifically positively stains cancer cells (A, C), a commercial antihuman IgG antibody showed extensive positive staining of lymphocytes, mesenchymal cells, and cancer cells in PDAC tissue (B) and of normal pancreatic cells in normal pancreatic tissue (D).

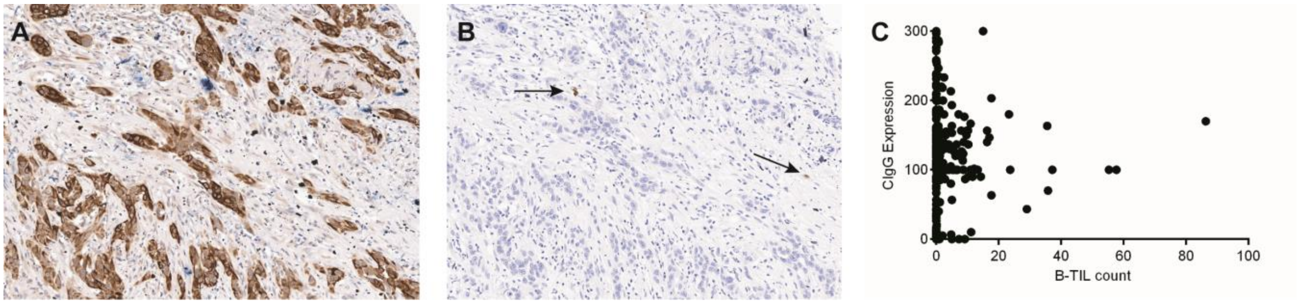

Fig. S2. Representative images of CIgG expression (A) and B-TILs (B) in a PDAC patient. Original magnification, 100×. (C) No significant correlation was observed between CIgG expression and the B-TIL count in PDAC (r_s=0.008, P=0.885). Spearman's rank correlation coefficients were employed due to the skewed distribution of the B-TIL count.

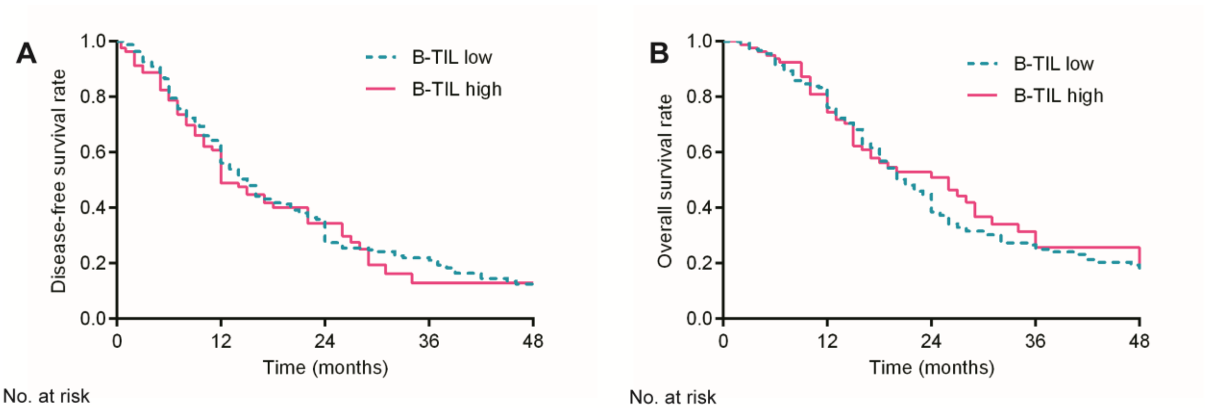

No. at risk

Low	246	150	60	27	14
High	80	46	17	4	3

No. at risk

Low	246	196	78	34	18
High	80	62	27	11	7

Fig. S3. Kaplan-Meier curves for disease-free survival (A) and overall survival (B) based on the B-TIL count in PDAC patients.

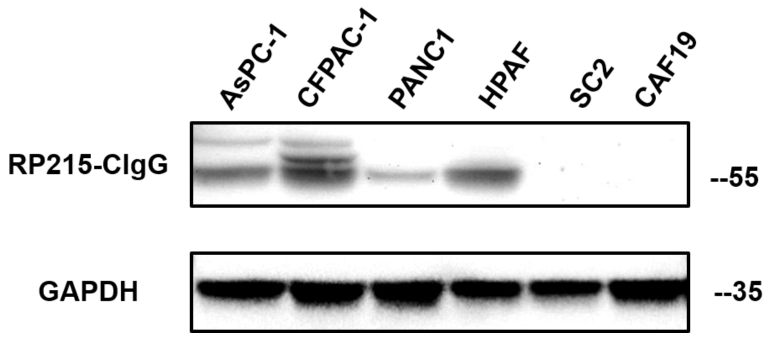

Fig. S4. CIgG expression in AsPC-1, CFPAC-1, PANC-1 and HPAF PDAC cell lines as well as fibroblast cell lines (SC2 and CAF19).
